# Modeling relationships between iron status, behavior, and brain electrophysiology: evidence from a randomized study involving a biofortified grain in Indian adolescents

**DOI:** 10.1186/s12889-022-13612-z

**Published:** 2022-07-06

**Authors:** Michael J. Wenger, Laura E. Murray Kolb, Samuel P. Scott, Erick Boy, Jere D. Haas

**Affiliations:** 1 Department of Psychology, Cellular and Behavioral Neurobiology, The University of Oklahoma, Norman, OK, USA; 2 Division of Nutritional Sciences, Cornell University, Ithaca, NY, USA; 3 Department of Nutrition Science, Purdue University, West Lafayette, IN, USA; 4 Poverty Health and Nutrition Division, International Food Policy Research Institute, Washington, DC, USA; 5 HarvestPlus, International Food Policy Research Institute, Washington, DC, USA

**Keywords:** Iron deficiency, Memory, Cognition, Brain, Electroencephalography

## Abstract

**Background:**

Iron deficiency (ID) and iron deficiency anemia (IDA) are highly-prevalent nutrient deficiencies and have been shown to have a range of negative effects on cognition and brain function. Human intervention studies including measures at three levels—blood, brain, and behavior—are rare and our objective was to model the relationships among measures at these three levels in school-going Indian adolescents.

**Methods:**

Male and female adolescents in rural India were screened for ID/IDA. Subjects consumed 2 meals/day for 6 months; half were randomly assigned to consume meals made from a standard grain (pearl millet) and half consumed meals made from an iron biofortified pearl millet (BPM). Prior to and then at the conclusion of the feeding trial, they completed a set of cognitive tests with concurrent electroencephalography (EEG).

**Results:**

Overall, serum ferritin (sFt) levels improved over the course of the study. Ten of 21 possible measures of cognition showed improvements from baseline (BL) to endline (EL) that were larger for those consuming BPM than for those consuming the comparison pearl millet (CPM). Critically, the best model for the relationship between change in iron status and change in cognition had change in brain measures as a mediating factor, with both change in serum ferritin as a primary predictor and change in hemoglobin as a moderator.

**Conclusions:**

A dietary intervention involving a biofortified staple grain was shown to be efficacious in improving blood iron biomarkers, behavioral measures of cognition, and EEG measures of brain function. Modeling the relationships among these variables strongly suggests multiple mechanisms by which blood iron level affects brain function and cognition.

**Trial registration:**

Registered at ClinicalTrials.gov, NCT02152150, 02 June 2014.

**Supplementary Information:**

The online version contains supplementary material available at (10.1186/s12889-022-13612-z).

## Introduction

Iron deficiency (ID) and iron deficiency anemia (IDA) are highly-prevalent nutrient deficiencies that can be found at high rates in developing and developed countries [1]. They have been shown to have negative effects on physical performance [2, 3] and work productivity [4, 5], and there is accumulating evidence that ID, without anemia, has a range of negative effects on cognition and brain function [6–11], including evidence that ID has a significant negative impact on academic performance [12]. However, the mechanisms by which ID exerts its negative impact in humans are not completely clear. In part this is because (a) only a small number of studies have collected both behavioral and neurophysiological data, and (b) an even smaller subset of those studies have attempted to model the hypothesis that changes in brain function mediate the relationships between changes in iron status and changes in behavioral measures of cognition.

Two aspects of brain function seem likely as mechanisms for ID-related cognitive deficits in otherwise healthy children and adults. The first is the effect of variations in iron levels on the neurotransmitter dopamine (DA), in particular the DA D2 receptor and DA transporter (DAT) in animal models [13–15]. Although there have been no direct measurements of the effects of ID or IDA on DA in humans, there is suggestive indirect evidence, including reductions in spontaneous blink rates in infants with IDA [16], with blink rates being related to levels of central DA [17]. Furthermore, there is evidence from both disease states and pharmacological manipulations that variations in DA levels have distinct effects on controlled attention and memory [18–20]. The second candidate is oxygen transport, given the importance of iron’s role in binding oxygen for transport via hemoglobin. This is underscored by the fact that the brain’s oxygen demands are quite high and that task-dependent changes in cerebral blood flow seem to reflect a generalized mobilization of brain resources [21–23]^1^.

In the present study, our measures of cognition included change in a set of behavioral tasks, including measures of controlled attention and memory, that we have shown to be sensitive to variations in iron status in adolescents and women of reproductive age [6, 8, 9]. Our measures of mediating brain function included a set of electroencephalographic (EEG) features that have been shown to be related to aspects of attention and memory retrieval, including power in the *α* and *γ* frequency bands [24, 25], and the amplitude of the N1 and P3 components [10, 11]. As our primary measure of iron status, we used serum ferritin (sFt), since our primary interest was in the effects of ID absent anemia and given the role of sFt in DA metabolism [26–28]. However, we also used hemoglobin (Hb) as a moderator, given the known demands that brain activity makes on oxygen use.

The purposes of the present study were the following. First, we sought to demonstrate that it is possible to obtain improvements in both brain function (as measured by EEG) and cognition in male and female adolescents in India following consumption of an iron-biofortified staple grain. Second, we sought to determine whether the EEG features related to attention and memory would be those that would mediate the relationship between improvements in blood iron biomarkers and behavioral measures of cognition. Third, we sought to determine whether Hb would be a mediator in the relationship between systemic iron status as measured by sFt and cognition. We should note that this is a secondary analysis of data from previously published randomized controlled trial (RCT) [9, 29]. Here we aim to test for plausibility of previous research results and investigate possible (hypothesized) neural mechanisms for how changes in iron status (the RCT primary outcome) affects behavior (the RCT secondary outcomes).

## Methods

This study was conducted among a subset of participants in a larger study of the efficacy of a dietary intervention. The parent study involved school children from economically disadvantaged families attending boarding schools in Ahmednagar district, Maharashtra, India, a rural community within a two-hour drive of Ahmednagar city. In this double-blind randomized efficacy trial, subjects (school children, 12–16 y) were stratified by hostel of residence and randomly assigned to receive either iron-biofortified pearl millet (BPM; variety ICTP8203, 86 ppm iron, n = 122) or a popular variety of pearl millet used for comparison (CPM; variety DG9444, 22 ppm iron, n = 124) from baseline to four months and variety JKBH778 (52 ppm iron) from four to six months. Because of higher-than-expected consumption, the CPM supply was exhausted after four months, and the original CPM variety DG9444 was replaced with variety JKBH778 available in the local market. Post-hoc analysis revealed that JKBH778 had intermediate iron content. All children received approximately 150 to 300 grams (dry) of the selected pearl millet variety daily in the form of a flat bread (bhakri) during lunch and dinner. Consumption was ad libitum. Bhakri was prepared twice daily by seven cooks who used only one type of pearl millet flour, and followed a protocol to standardize bhakri diameter, weight and consistency. Iron status (Hb, serum ferritin [sFt], soluble transferrin receptor [sTfR], calculated body iron [BdFe] [34]), inflammation (C-reactive protein [CRP], *α*-1 acid glycoprotein [AGP]), and anthropometric indices were evaluated at enrollment, four months, and six months. This trial was registered at ClinicalTrials.gov, NCT02152150, 02 June 2014. Complete details of the study design and laboratory analyses can be found in [9, 29].

### Subjects

**Fig. 1:**
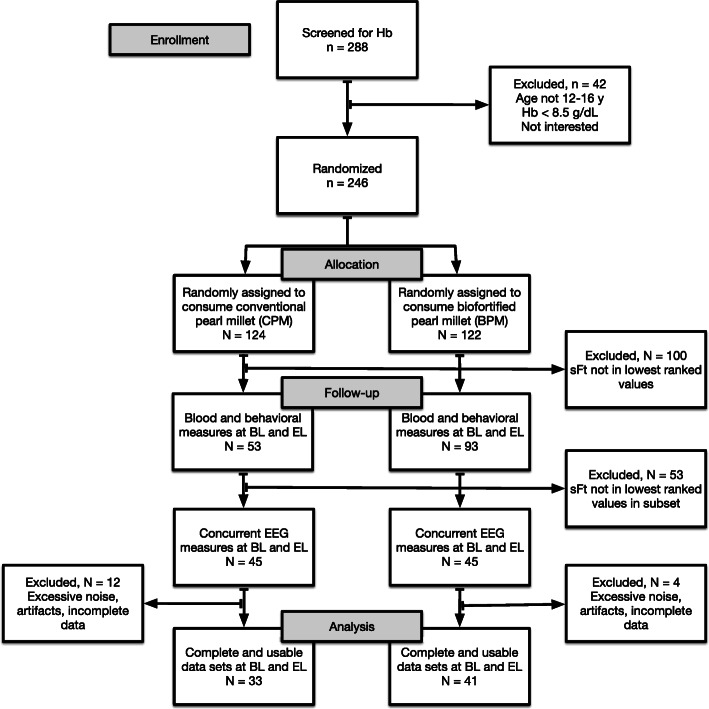
CONSORT diagram for the study

Subjects were female and male students (12–16 y) attending a rural boarding school. The school was selected based on the high prevalence of anemia (> 25%) found in a prescreening survey and on its capacity to support the efficacy trial. Subjects had to be in good health without chronic disease or acute illness, not taking iron supplements or medications that would interfere with iron absorption, residing full-time at the boarding school, and not severely anemic; those with Hb levels <85 g/L were ineligible and were referred to a physician for follow-up. Iron deficiency was defined as sFt levels <20 *μg*/*L* [35, 36] and anemia was defined as Hb levels <13 for males aged 15–16 years and <12 *μg*/*dL* for all others. Of the 288 subjects screened (see Fig. 1), 42 were ineligible and 246 were enrolled in the parent feeding trial in September 2011. Anthelminthic treatment (200 mg albendazole) was provided to subjects four weeks before the baseline assessment and at the study midpoint. A subset of 146 subjects was chosen to undergo functional testing—including tests of both physical (not described here) and cognitive function—based on having the lowest overall ranked screening sFt levels, as it was thought that these individuals would have the greatest potential to benefit from the intervention and demonstrate cognitive change. Of these, a subset of 90 were selected for cognitive testing with concurrent EEG, with equal numbers in each of the two treatment groups; selection was again determined by having the lowest ranked sFt levels; given the low prevalence of inflammation (defined as AGP >1.0 or CRP >5.0), there was no need to correct the sFt measures. The minimum sample size estimated [37] to allow for 80% power and *α*=0.05 for differences in the amplitudes of event-related potentials, based on EEG data from Otero et al. [10] was 30 per group. To account for potential data loss and dropout, we increased the sample requirement by 50%, to 45 subjects per group. After removing subjects for whom usable EEG data (i.e., artifact- and noise-free) was not available at both baseline and endline, the final set of data submitted to analyses was composed of 33 subjects who consumed the comparison pearl millet and 41 subjects who consumed the biofortified pearl millet. This level of data loss is consistent with previous work, others [10] as well as our own [7, 35].

### Cognitive testing with concurrent EEG

Five cognitive/behavioral tasks—three attention tasks and two memory tasks—assessed aspects of cognition with hypothesized relationship to iron status; procedural details for each of the tasks are presented in the online supporting material (Additional file 1). Testing sessions lasted approximately 60 minutes. DMDX [38] software was used to run all of the tasks, all of which were developed and programmed by MJW. The tasks were presented on a set of Windows-based laptop computers with 36 cm (diagonal) displays, running at 2.5 GHz, with at least 4 Gb of RAM and at least 320 Gb of hard disk storage. Stimulus onsets were synchronized to the vertical refresh rate of the monitor and keyboard responses were timed to ±1 ms. Stimuli for the tasks were either grayscale images or white text on a black background (see example stimuli in Additional File 1). The tasks were presented in the following fixed order: 1) The Simple Reaction Time (SRT) task was the most basic task and assessed processing speed absent significant attentional or memory demands. Subjects responded with a single keypress to a symbol that appeared in the center of the screen. 2) The Go/No-Go (GNG) task provided an estimate of the effectiveness of sustained attention and the speed of simple attentional capture. Subjects were required to make a decision about one of two possible stimuli, a vertical or horizontal bar that were randomly assigned to be either a Go or a No-Go stimulus. Subjects were instructed to respond using a button press as quickly as possible when seeing the Go stimulus (which appeared 20% of the time) and not to respond when seeing the No-Go stimulus (which appeared 80% of the time). 3) The Attentional Network Task (ANT) was a modified flanker task assessing three distinct functions of attention: alerting (low-level attentional capture), orienting (mid-level spatial selective attention), and conflict (high-level selection or control [39]). On each trial, the participant was presented with either an informative or uninformative cue as to the location of an upcoming test stimulus display (above or below fixation) and was required to press a button to indicate whether a centrally-presented arrow in the display pointed to the left or right, while disregarding flanking elements (congruent, incongruent, or neutral distractors) on either side of the stimulus. Participants were instructed to respond as quickly as possible by pressing one key with their left index finger if the central arrow pointed left and another key with their right index finger if the central arrow pointed right. 4) In the Composite Face Effect (CFE) task, participants learned to identify (using two different buttons) two familiar (famous celebrities; familiarity was confirmed in a pilot test with similarly aged non-participants in the same school) and two unfamiliar faces. They were then shown images in which the top and bottom half of the face could be from the same or different faces and were either aligned or misaligned. Participants were instructed to respond only to the cued half of the face and to ignore the other half. 5) Finally, in the Cued Recognition Task (CRT), participants were first shown a set of 24 culturally appropriate pictures, one at a time for three seconds each, to commit to memory. Next, they viewed a set of 48 pictures, 24 from the memorized set and 24 new pictures. Participants were instructed to identify whether the picture was from the memorized set or not (i.e. was “old” or “new” to them). The amount of visual information available was varied by covering picture quadrants with black squares such that 50, 75 or 100 percent of any given picture in the second set of 48 was visible. This task was included due to evidence that the integrity of neural circuits supporting recognition memory, particularly those involving the hippocampus, is correlated with the amount of work that can be accomplished during recognition [35, 40].

Concurrent EEG data were acquired using a 32-channel geodesic electrode net connected to a 32-channel amplifier (EGI/Philips, Eugene, OR). EEG, as measured at the scalp, reflects the coordinated activity of spatially-adjacent populations of similarly-oriented neurons, propagated through the brain, skull, and skin [41]. Their activity corresponds to the computational operations that are needed to allow a stimulus to be transformed into a response. To the extent that these populations of neurons are effectively carrying out these computations, the amplitude and power of their conjoint activity will be higher, relative to any iron-dependent impairments (e.g., neurotransmitter signaling or oxygen transport). Data were digitized at 1 KHz during acquisition and down-sampled to 250 Hz for analyses. Impedances were maintained at ≤50 k *Ω* during acquisition. Preprocessing for statistical analysis is described in the Supplementary information.

### Statistical analyses

Differences in biomarkers at baseline were analyzed using a 2 (treatment group: CPM, BPM) ×2 (sex: female, male) analysis of variance (ANOVAs). Differences in prevalences of biological conditions (e.g., anemia) were analyzed using a *χ*^2^ test of association. The first set of analyses were intention-to-treat analyses of all the dependent measures. These analyses took the form of a difference-in-differences analysis, with the two factors being group (comparison vs. biofortified pearl millet) and assessment (BL vs. EL). Note that each of the dependent measures is a unique and independent assessment of a specific aspect of cognitive functioning, thus there was no need across the set of variables for any corrections for multiple comparisons. A second set of analyses were done to assess the plausibility of the change in iron status as causal in changes in the dependent variables across both treatment groups. These analyses were done by regressing change in the behavioral and EEG variables onto change in the set of iron status biomarkers, testing the hypothesis that adolescents who experienced a greater improvement in iron status would show greater improvement in their behavioral and brain measures. The set of candidate models included a “null” model (intercept only); a full model, in which all allowable independent predictors (iron biomarkers) were included (i.e., sFt and sTfR were not allowed in a model including BdFe); and a model whose form was determined by step-wise model selection procedures to minimize the number of parameters while maximizing *R*^2^; age and sex were included as potential covariates. We selected a best model using the criteria that the model had to provide a better account than the null model, than any of the competing models (as assessed using the Akaike Information Criterion [42]), and account for at least 10% of the variance. Age and sex were included as covariates in the final model only if they were shown to be statistically significant.

The third set of analyses involved estimating a set of mediation models incorporating moderator variables [43] to represent the hypothesis that changes in brain activity mediate the relation between changes in peripheral iron status and changes in behavior. These analyses were performed on three composite (*Z*-transformed and scaled such that positive values indicate improvement from baseline to endline) behavioral variables: change in low-level attentional capture, change in high-level attentional selection, and change in efficiency of memory retrieval. The attentional capture variable was formed by averaging the *Z*-transformed change in reaction time (RT) in the GNG and the change in RT in the 2-cue condition in the ANT. The attentional selection variable was formed by averaging the *Z*-transformed change in RT in the spatial cue and inconsistent flanker conditions of the ANT. The memory efficiency variable was formed by averaging the *Z*-transformed change in RT and sensitivity measures in the CFE and the percentage change in capacity (PCC) in the CRT. Three sets of mediating variables were specified for these three composite behavioral variables, based on the regression analyses performed to assess plausibility. The mediating variable for attentional capture was a composite (averaged *Z*-score) of the N1 and P3 amplitudes for the corresponding conditions for the behavioral variables. The mediating variable for attentional selection was a composite of the N1 and P3 amplitudes for the corresponding behavioral conditions. The mediating variable for memory retrieval efficiency was a composite of the N1 and P3 amplitudes and *γ*-band power for the corresponding behavioral conditions. The choice of variables comprising the composite mediators was determined by the results of the regression analyses conducted to assess plausibility that change in iron status was the source of change in the EEG variables. The possible predictors/moderators were the normalized (Z-transformed) change in Hb, sFt, and sTfR, based on the fact that these were the only measures that were identified as significant predictors in the plausibility analyses. These specific variables and composites were selected on the basis of a stepwise procedure which began with all possible variables, and proceeded by removing variables whose parameters were not reliably different from zero (0) and then refitting, and repeating this until we found the smallest set of variables that could account for the greatest amount of variance.

These variables were used to fit candidate mediation models with effect modifiers for each behavioral composite score, using all possible combinations of the EEG composite scores as both mediators and effect modifiers. The final set of variables was determined by first fitting the models with the complete set of variables and then deleting those that did not reach criterion for statistical significance. In addition, a “direct effect only” model and a “scrambled” model were fit to the data for each composite behavioral score. The “direct effect only” model did not include any mediators or effect modifiers, and the “scrambled” model was a model created by rearranging the order of the effects in the best-fitting model. The “best” model met the following criteria: (a) the overall *F*-statistic for the model had to be statistically significant; (b) all component *R*^2^ values needed to be ≥0.10; (c) all values for model parameters (excluding the intercept) needed to be significantly different from zero; and, (d) based on *R*^2^ and AIC values, the “best” model had to outperform all alternative models, including the “direct effect only” and “scrambled” models.

## Results

### Sample characteristics

Characteristics of the sample at baseline are presented in Table 1, separately for each of the treatment conditions. Supplementary Table 1 in the supplemental information (Additional file 1) further breaks down these descriptives in terms of sex. Note that these comparisons are intended only to characterize the sample at BL and are not intended to generalize to the population. In terms of the iron biomarkers, the only significant difference was a main effect due to sex for Hb levels, with females overall having lower Hb levels than males. There were no reliable effects due to assignment to treatment condition. There were reliable effects due to sex in terms of prevalence of anemia, IDA, and iron deficiency without anemia (iron deficient non-anemic, IDNA), with higher prevalence in females than in males for anemia and IDA, the opposite ordering for IDNA.

**Table 1:** Baseline characteristics of the sample. Presented are means (standard errors) and *N*s (percentage) for the groups consuming the comparison and the biofortified pearl millet

		Comparison	Biofortified
	Overall	n = 33	n = 41
Age (y)	13.5 (0.2)	13.58 (0.22)	13.44 (0.20)
Hb, g/dL	12.13 (0.13)	12.18 (0.19)	12.09 (0.19)
sFt, ng/mL	15.41 (1.56)	13.38 (1.69)	17.05 (2.45)
sTfR, ug/mL	7.13 (0.59)	7.35 (0.71)	6.98 (0.40)
BdFe (mg/kg)	0.61 (0.37)	0.31 (0.48)	0.86 (0.55)
*Prevalences*			
Anemia	26 (35)	11 (33)	15 (37)
sFt <15.0	46 (62)	23 (70)	23 (56)
sTfR >8.3	11 (15)	3 (9)	8 (20)
BdFe <0.0	26 (35)	12 (36)	14 (34)
IDA	16 (22)	8 (24)	8 (20)
IDNA	30 (41)	15 (45)	15 (37)
Inflammation	4 (5)	1 (3)	3 (7)

### Intention-to-treat analyses

Results of the difference-in-differences analyses of the blood iron biomarkers and behavioral variables are presented in Table 2. The only difference to reach significance for the iron biomarkers was the difference due to assessment (EL vs. BL) for sFt. For the behavioral variables, four of 21 possible differences due to condition were significant, with the difference favoring BPM. Fifteen of 21 possible differences due to assessment were significant, with the difference favoring EL. Finally, ten of 21 possible differences in differences were significant, with change from BL to EL being larger for BPM than for CPM.

**Table 2:** Difference-in-differences analyses of the blood and behavioral variables. Entries are least-squares means (95% confidence intervals). Entries in bold are those for which the confidence interval does not contain 0. Note: CPM = comparison pearl millet, BPM = biofortified pearl millet

		Baseline		Endline		Difference in		
Task	Variable	CPM	BPM	CPM	BPM	Condition	Assessment	Differences
*Blood variables*								
	Hb (g/dL)	12.18 (11.79, 12.57)	12.09 (11.75, 12.44)	12.11 (11.72, 2.50)	12.41 (12.06, 12.76)	0.09 (-0.43, 0.61)	0.32 (-0.17, 0.81)	-0.39 (-1.13, 0.34)
	sFt (ng/mL)	13.38 (9.24, 17.51)	17.05 (13.34, 20.76)	14.93 (10.79, 19.07)	22.31 (18.60, 26.03)	-3.67 (-9.24, 1.89)	**5.26 (0.01, 10.51)**	-3.70 (-11.60, 4.16)
	sTfR (ug/mL)	7.35 (6.48, 8.22)	6.99 (6.21, 7.76)	7.92 (7.05, 8.79)	6.90 (6.12, 7.68)	0.36 (-0.80, 1.53)	-0.08 (-1.18, 1.02)	0.65 (-0.99, 2.30)
	BdFe (mg/kg)	0.31 (-0.76, 1.38)	0.86 (-0.10, 1.82)	0.49 (-0.58, 1.56)	1.72 (0.76, 2.68)	-0.54 (-1.98, 0.90)	0.86 (-0.50, 2.22)	-0.69 (-2.72, 1.35)
*Behavioral variables*								
SRT	RT (ms)	1186 (1154, 1218)	1181 (1153, 1210)	1051 (1018, 1083)	1082 (1053, 1111)	4 (-39, 48)	**-100 (-141, -59)**	-36 (-97, 26)
GNG	RT (ms)	627 (600, 654)	661 (637, 684)	592 (565,619)	546 (522, 569)	-34 (-69, 2)	**-115 (-148, -82)**	**80 (30, 130)**
ANT	RT 0 cues (ms)	658 (624, 692)	677 (647, 707)	668 (634, 702)	692 (662, 723)	-19 (-64, 27)	15 (-28, 58)	-5 (-69, 59)
	RT 2 cues (ms)	633 (597, 668)	712 (680, 745)	632 (596, 668)	581 (549, 613)	**-80 (-128, -32)**	**-131 (-177, -86)**	**130 (62, 199)**
	RT alerting (ms)	22 (-10, 54)	-36 (-65, -7)	44 (12, 76)	111 (82, 140)	**58 (15, 101)**	**147 (106, 187)**	**-125 (-186, -64)**
	RT center cues (ms)	669 (634, 703)	687 (657, 718)	618 (583, 652)	643 (613, 674)	-19 (-65, 27)	**-44 (-87, -1)**	-6 (-71, 58)
	RT spatial cues (ms)	575 (548, 602)	641 (617, 665)	614 (587, 641)	561 (537, 585)	**-66 (-102, -30)**	**-79 (-113, -45)**	**118 (67, 169)**
	RT orienting (ms)	90 (54, 125)	46 (15, 78)	12 (-24, 47)	82 (50, 114)	44 (-4, 91)	35 (-10, 80)	**-114 (-181, -46)**
	RT cons flankers (ms)	677 (646, 709)	692 (664, 720)	643 (612, 675)	686 (658, 715)	-15 (-57, 28)	-5 (-45, 34)	-29 (-88, 31)
	RT incons flankers (ms)	766 (731, 801)	814 (742, 846)	814 (778, 849)	725 (693, 756)	**-48 (-95, -1)**	**-89 (-134, -44)**	**137 (70, 204)**
	RT conflict (ms)	89 (64,114)	121 (99, 144)	170 (145, 195)	38 (16, 61)	-32 (-66, 1)	**-83 (-115, -51)**	**165 (117, 212)**
CFE	CFE RT (ms)	59 (28, 90)	39 (11, 67)	47 (16, 78)	92 (64, 120)	20 (-22, 61)	**53 (13, 92)**	**-64 (-123, -5)**
	CFE hit rate (ms)	-0.02 (-0.07, 0.03)	0.01 (-0.03, 0.06)	-0.01 (-0.06, 0.04)	0.03 (-0.01, 0.07)	-0.04 (-0.10, 0.03)	0.02 (-0.04, 0.08)	-0.01 (-0.09, 0.09)
	CFE false alarm rate (propn)	-0.03 (-0.07, 0.02)	0.03 (-0.01, 0.08)	0.01 (-0.03, 0.06)	-0.04 (-0.07, 0.01)	-0.06 (-0.12, 0.01)	**-0.06 (-0.12, -0.01)**	**0.10 (0.01, 0.19)**
	CFE sensitivity (SD)	0.05 (-0.21, 0.31)	0.09 (-0.14, 0.32)	0.08 (-0.18, 0.34)	0.42 (0.19, 0.65)	-0.04 (-0.38, 0.31)	**0.33 (0.01, 0.66)**	-0.30 (-0.79, 0.19)
	CFE bias (SD)	0.03 (-0.11, 0.16)	0.08 (-0.04, 0.20)	-0.01 (-0.14, 0.14)	-0.10 (-0.22, 0.03)	-0.06 (-0.24, 0.13)	**-0.18 (-0.35, -0.01)**	0.15 (-0.11, 0.41)
CRT	RT, new items (ms)	939 (873, 1006)	862 (803, 922)	853 (787, 919)	879 (819, 938)	77 (-12, 166)	16 (-67, 100)	-103 (-228, 23)
	RT, old items (ms)	737 (698, 777)	720 (684, 755)	747 (707, 787)	666 (630, 702)	18 (-36, 71)	**-54 (-104, -3)**	64 (-12, 139)
	Sensitivity (SD)	3.26 (2.93, 3.58)	3.32 (3.03, 3.62)	3.35 (3.03, 3.68)	3.93 (3.64, 4.23)	-0.07 (-0.50, 0.37)	**0.61 (0.20, 1.02)**	-0.52 (-1.13, 0.10)
	Bias (SD)	0.02 (-0.14, 0.18)	-0.01(-0.15, 0.14)	0.05 (-0.11, 0.21)	-0.08 (-0.22, 0.06)	0.03 (-0.19, 0.24)	-0.08 (-0.27, 0.13)	0.10 (-0.20, 0.40)
	Pct change in capacity (%)	31 (23, 40)	35 (28, 42)	52 (45, 60)	80 (73, 87)	-4 (-14, 7)	**45 (35, 55)**	**-24 (-39, -9)**

The results of the difference-in-difference analyses of the EEG variables are presented in Table 3. None of the 20 possible differences due to condition were significant. Five of the 20 possible differences due to assessment were significant, with the difference favoring EL over BL. Finally, only one of the 20 possible difference-in-differences was significant, with change from BL to EL being larger for BPM than for CPM.

**Table 3:** Difference-in-differences analyses of the EEG variables. Entries are least-squares means (95% confidence intervals). Entries in bold are those for which the confidence interval does not contain 0. Note: CPM = comparison pearl millet, BPM = biofortified pearl millet

		Baseline		Endline		Difference in		
Task	Variable	CPM	BPM	CPM	BPM	Condition	Assessment	Differences
SRT	N1 amplitude (uV)	-2.83 (-3.74, -1.92)	-2.06 (-2.89. -1.23)	-1.12 (-2.03, -0.21)	-5.75 (-6.60, -4.93)	-0.77 (-2.00, 0.46)	**-3.69 (-4.85, -2.52)**	**5.40 (3.66, 7.14)**
	P3 amplitude (uV)	1.78 (1.35, 2.20)	1.59 (1.20, 1.97)	1.48 (1.05, 1.91)	2.08 (1.69, 2.46)	0.19 (-0.38, 0.76)	0.49 (-0.05, 1.03)	-0.78 (-1.59., 0.03)
	Alpha power (db/Hz)	0.249 (0.246, 0.253)	0.251 (0.248, 0.254)	0.254 (0.251, 0.257)	0.251 (0.248, 0.254)	-0.002 (-0.006, 0.003)	0.000 (-0.004,0.004)	0.004 (-0.002, 0.011)
	Gamma power (db/Hz)	0.047 (0.044, 0.051)	0.047 (0.044, 0.051)	0.048 (0.044, 0.051)	0.052 (0.048, 0.056)	0.000 (-0.005, 0.005)	0.005 (0.000, 0.010)	-0.005 (-0.012, 0.003)
GNG	N1 amplitude (uV)	-1.82 (-2.51, -1.14)	-1.46 (-2.05, -0.86)	-0.72 (-1.40, -0.04)	-1.31 (-1.90, -0.71)	-0.37 (-1.27, 0.54)	0.15 (-0.70, 0.99)	0.96 (-0.33, 2.24)
	P3 amplitude (uV)	1.89 (1.25, 2.52)	1.42 (0.87, 1.98)	0.81 (0.17, 1.44)	1.13 (0.58, 1.69)	0.46 (-0.38, 1.31)	-0.29 (-1.08, 0.49)	-0.79 (-1.98, 0.40)
	Alpha power (db/Hz)	-3.96 (-7.83, -0.08)	-3.80 (-7.57, -0.03)	-4.51 (-8.38, -0.63)	-4.14 (-7.91, -0.37)	-0.16 (-5.57, 5.25)	-0.34 (-5.67, 5.00)	-0.21 (-7.86, 7.44)
	Gamma power (db/Hz)	-17.64 (-22.37, ’12.90)	-17.32 (-22.32, -12.32)	-41.22 (-45.96, -36.48)	-40.94 (-45.94, -35.94)	-0.32 (-7.20, 6.57)	**-23.62 (-30.69, -16.55)**	0.04 (-9.70, 9.78)
ANT	N1 amplitude (uV)	-2.28 (-2.57, -2.00)	-2.33 (-2.59, -2.06)	-2.00 (-2.27, -1.71)	-2.33 (-2.59, -2.06)	0.05 (-0.34, 0.43)	-0.12 (-0.50, 0.25)	0.41 (-0.14, 0.96)
	P3 amplitude (uV)	2.61 (2.37, 2.86)	2.75 (2.52, 2.98)	2.06 (1.81, 2.30)	2.61 (2.38, 2.83)	-0.14 (-0.47, 0.20)	-0.14 (-0.46, 0.18)	-0.41 (-0.89, 0.06)
	Alpha power (db/Hz)	-3.96 (-7.84, -0.08)	-3.80 (-7.57, -0.03)	-4.51 (-8.38, -0.63)	-4.14 (-7.91, -0.37)	0.16 (-5.57, 5.25)	-0.34 (-5.67, 5.00)	-0.21 (-7.86, 7.44)
	Gamma power (db/Hz)	-17.64 (-22.37, -12.90)	-17.32 (-22.32, -12.32)	-41.22 (-45.96, -36.48)	-40.94 (-45.94, -35.94)	-0.32 (-7.20, 6.60)	**-23.62 (-30.69, -16.55)**	0.04 (-9.70, 9.78)
CFE	N1 amplitude (uV), contrast	-1.50 (-0.99, -2.00)	-1.23 (-0.77, -1.69)	-2.41 (-1.91, -2.92)	-2.99 (-2.53, -3.45)	-0.27 (-0.41, -0.96)	**-1.76 (-1.11, -2.41)**	-0.85 (-1.81, 0.12)
	P3 amplitude (uV), contrast	1.52 (1.12, 1.91)	1.82 (1.47, 2.18)	1.48 (1.09, 1.87)	2.05 (1.69, 2.40)	-0.31 (-0.84, 0.22)	0.22 (-0.28, 0.73)	-0.26 (-1.01, 0.49)
	Alpha power (db/Hz), contrast	3.01 (2.33, 3.70)	3.33 (2.66, 4.00)	3.00 (2.32, 3.68)	2.90 (2.23, 3.58)	-0.32 (-1.27, 0.64)	-0.43 (-1.37, 0.52)	0.42 (-0.94, 1.77)
	Gamma power (db/Hz), contrast	3.51 (2.82, 4.20)	3.11 (2.38, 3.84)	2.52 (1.82, 3.22)	3.00 (2.28, 3.73)	0.40 (-0.60, 1.40)	-0.10 (-1.13, 0.93)	-0.89 (-2.31, 0.54)
CRT	N1 amplitude, slope (uV)	0.10 (-0.24, 0.43)	-0.10 (-0.39, 0.19)	0.24 (-0.10, 0.58)	-0.12 (-0.42, 0.17)	0.19 (-0.25, 0.64)	-0.03 (-0.44, 0.39)	0.17 (-0.46, 0.80)
.	P3 amplitude, slope (uV)	0.42 (-0.05, 0.88)	0.38 (-0.02, 0.79)	0.57 (0.11, 1.03)	0.93 (0.52, 1.34)	0.03 (-0.58, 0.65)	0.55 (-0.03, 1.13)	-0.40 (-1.27, 0.47)
	Alpha power, slope (db/Hz)	-0.58 (-1.28, 0.13)	-0.18 (-0.44, 0.80)	-0.24 (-0.94, 0.46)	0.63 (0.01, 1.25)	-0.76 (-1.70, 0.18)	0.45 (-0.42, 1.33)	-0.12 (-1.44, 1.21)
	Gamma power, slope (db/Hz)	-0.59 (-1.64, 0.46)	0.26 (-0.67, 1.18)	0.25 (-0.80, 1.30)	2.22 (1.29, 3.15)	-0.84 (-2.24, 0.56)	**1.97 (0.66, 3.28)**	-1.13 (-3.11, 0.85)

**Fig. 2:**
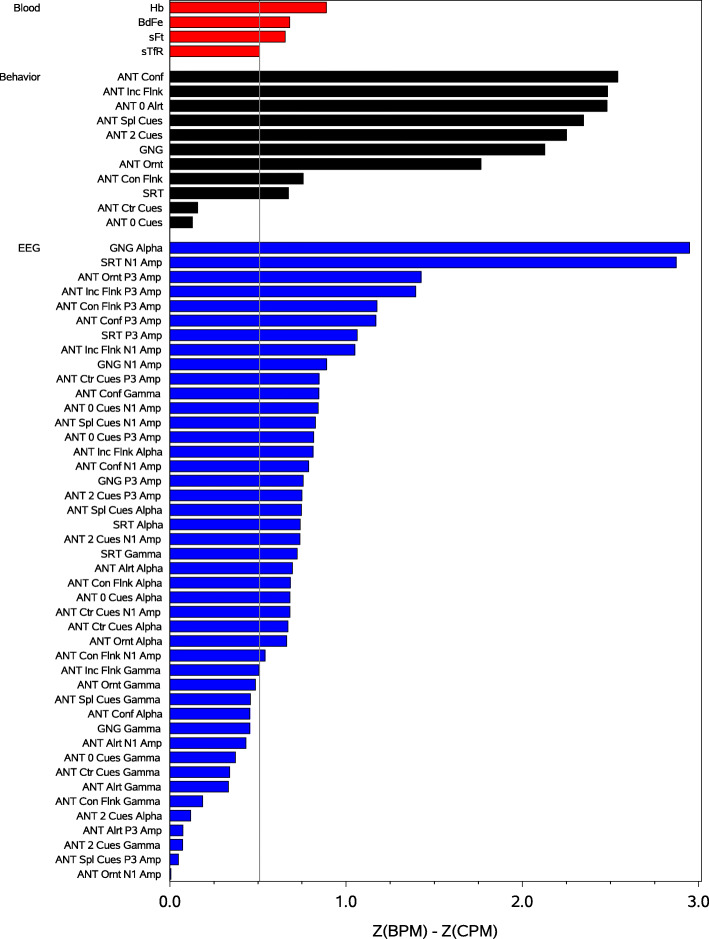
Magnitude of the difference in the normalized amount of change observed for those consuming the biofortified pearl millet (BPM) and those consuming the control pearl millet (CPM), on each of the blood, behavior, and EEG variables for the attention tasks. The reference lines denote the smallest amount of change observed for the blood biomarkers

**Fig. 3:**
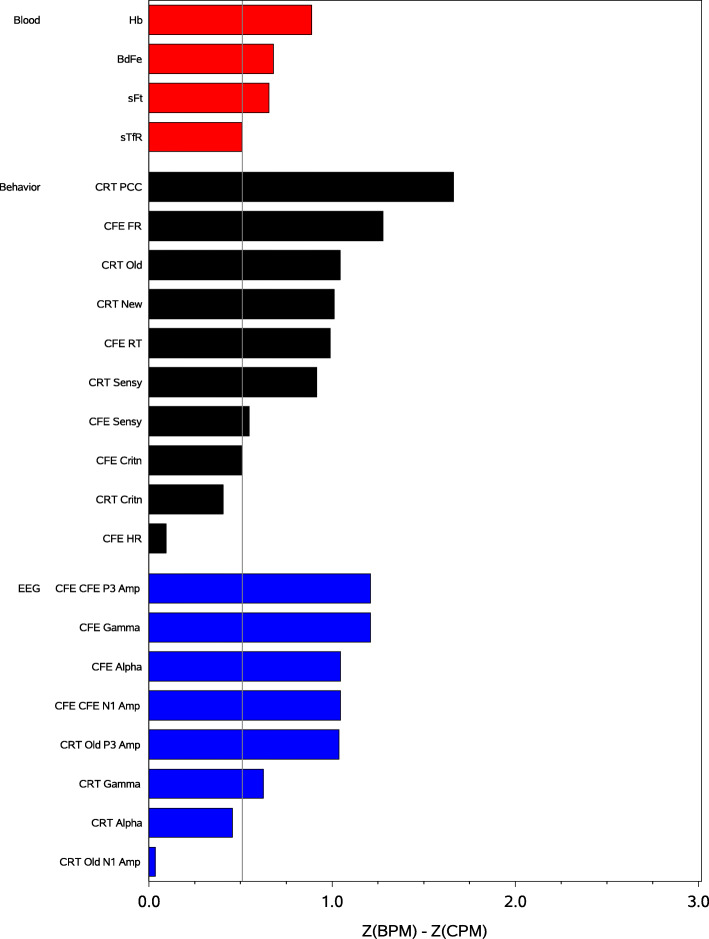
Magnitude of the difference in the normalized amount of change observed for those consuming the biofortified pearl millet (BPM) and those consuming the control pearl millet (CPM), on each of the blood, behavior, and EEG variables for the memory tasks. The reference lines denote the smallest amount of change observed for the blood biomarkers

A summary of the amount of change observed for all of the dependent variables is plotted as the difference between the normalized (Z-unit) change for the BPM group and normalized change for the CPM group in Figs. 2 and 3; Fig. 2 plots the results for the attentional variables and Fig. 3 plots the results for the mnemonic variables. Note that, in all cases for all variables, change was greatest for those consuming the BPM (as indicated by all of the difference scores being >0. Also note that, relative to the smallest amount of positive change observed for the blood variables, the magnitude of the positive difference was larger for the majority of the behavioral and EEG variables.

### Plausibility analyses

The results of the regression analyses performed to assess the plausibility that changes observed in the behavioral and the EEG variables were related to changes in blood parameters are reported in Table 4. For the behavioral variables, all of the tasks except for the SRT had at least one variable for which the regression met our criteria for sufficiency. Of the 16 behavioral variables for which this was true, 13 of the cases involved sFt or BdFe as the best predictor, with two of the remaining cases having Hb as the best predictor and one having sTfR as the best predictor^2^. For those variables for which a model met our criteria, the average proportion of variance accounted for was 0.17. With respect to the EEG variables, all of the tasks had at least one variable for which a model met our sufficiency criteria. In those cases, either sFt or BdFe were selected as the best predictor in all but one case, in which Hb was selected as the best predictor. For those variables that had a model that met our sufficiency criteria, the average proportion of variance accounted for was 0.16.

**Table 4:** Results of the regression analyses examining change in the behavioral and EEG variables as a function of change in the blood iron biomarkers (analyses for plausibility). Note that if a variable does not appear in this table, no acceptable regression model was identified for that variable

		Change in Blood Variable			
Task	Change variable	Predictor	Intercept	$\hat {\beta }$	*R*^2^
*Behavioral variables*					
GNG	MedianRT	BdFe	64	20	0.21
ANT	RT, 2 cues	Hb	55.8	22.1	0.17
		sFt		3.9	
	Alerting	sFt	81.0	4.2	0.19
	RT, spatial cues	sFt	8.7	4.0	0.18
	Orienting	sFt	-36.5	4.3	0.14
	RT, inconsistent flankers	Hb	-14.6	20.5	0.26
		sFt		4.2	
	Conflict	sFt	-0.51	3.2	0.31
		sTfR		-83.1	
CFE	RT	BdFe	6.86	17.12*	0.10
	Sensitivity	BdFe	0.13	0.16	0.16
	Bias	BdFe	-0.03	0.07	0.11
CRT	RT, new items	BdFe	47	-32	0.11
	RT, old items	BdFe	44	-23	0.15
	PCC	sFt	30.00	0.78	0.11
*EEG variables*					
SRT	N1 amplitude	Hb	0.86	0.95	0.11
		BdFe		0.46	
GNG	P1 amplitude	sFt	-1.4	0.08	0.13
	N! amplitude	sFt	-0.93	0.11	0.35
ANT	N1 amplitude, 2 cues	sFt	1.61	0.05	0.10
	N1 amplitude, orienting	sFt	-1.23	0.09	0.21
	P3 amplitude, orienting	sFt	-1.03	0.03	0.10
	P3 amplitude, incongruent flankers	sFt	1.78	0.06	0.25
	N1 amplitude, conflict	sFt	-0.49	0.05	0.23
	Gamma power, 2 cues	sFt	-1.02	0.05	0.15
CFE	Interaction contrast, N1 amplitude	sFt	1.01	0.05	0.15
CRT	P3 amplitude, old items 4 cues	sFt	1.24	0.03	0.10
	Gamma power, old items, 4 cues	sFt	5.49	0.11	0.10
	Gamma slope, old items 4 cues	sFt	1.55	0.07	0.12

### Mediation analyses

**Fig. 4:**
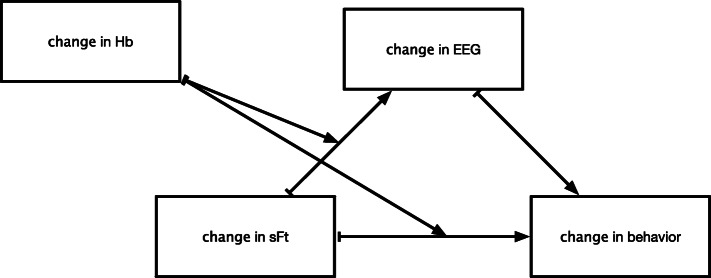
Common form for the best-fitting mediation models with effect modifiers. Change refers to change from baseline to endline. In all cases (see Table 5), all parameters were positive

The common form for the selected mediation models for each of the three composite variables is presented in Fig. 4, and the estimated parameters for each model are presented in Table 5. For all three composite variables, change in sFt was selected as the primary predictor and change in Hb was selected as an effect modifying variable. Specifically, the effect of a change in sFt on change in behavior was mediated by a change in brain activity. In addition, both the direct relationship between change in sFt and change in behavior and the relationship between change in sFt and change in brain activity was modified by change in Hb.

**Table 5:** Estimates of the direct and indirect effects of each of the mediation models for change in behavior

				Effects	
Predictor	Mediator	Moderator	Outcome	Direct	Indirect
*Δ* sFt	*Δ* EEG	*Δ* Hb	*Δ* attentional capture	0.1882 (-0.0484, 0.4247)	0.1183 (0.0221, 0.3547)
*Δ* sFt	*Δ* EEG	*Δ* Hb	*Δ* attentional selection	0.0379 (-0.1387, 0.2145)	0.1234 (0.0437, 0.2031)
*Δ* sFt	*Δ* EEG	*Δ* Hb	*Δ* memory efficiency	0.1242 (-0.0749, 0.3233)	0.1930 (0.0150, 0.3441)

## Discussion

The literature on the functional effects of ID and IDA has an increasing number of examples documenting that ID and IDA extract significant costs in terms of cognition [6, 8, 12, 44–46]. The present study is among the first to depart from a descriptive approach to move towards tests of hypotheses regarding mechanisms. We focused on controlled attention and memory, as these are functions that have been shown to be dependent on the integrity of the dopaminergic system, and work in animal models have pointed to significant effects of ID/IDA on the dopaminergic system. In addition, the study is among the first to both collect simultaneous behavioral and EEG data and to explicitly model the mediating role that changes in brain function have in relating changes in blood iron biomarkers to changes in behavior. Finally, the present study is among the first to explicitly model the hypothesis that changes in oxygen transport capacity (as measured by changes in Hb) work in conjunction with changes in neurotransmitters (as suggested by changes in sFt).

We demonstrated that changes in blood iron biomarkers were associated with reliable changes in both behavioral and EEG features associated with a variety of aspects of attention. These included changes in power in the *α* and *γ* frequency bands and, most regularly, the amplitude of the N1 component. This suggests the plausibility of the hypothesis that ID produces negative changes in dopaminergic regulation, particularly in basal ganglia, and that repletion corrects this, with the outcome measurable as a function of the output of basal ganglia circuits to cortex, and replicates a similar set of results obtained with a population of college-aged women consuming an iron biofortified bean [35].

We also demonstrated, by way of statistical modeling of the relationships among the three classes of variables (blood iron biomarkers, ERP data, and behavioral measures), that the negative effects of iron deficiency with and without anemia on cognition may also be a function of deficits in oxygen transport. A sizable minority (35%) of the participants in this study had Hb levels below the criterion for anemia. Consequently, it is perhaps not surprising that the statistical modeling of the relationships among the blood, brain, and behavioral variables showed that change in Hb needed to be included as a positively-signed moderator in order to provide the best description of the data, even though Hb played a minor role in the plausibility analyses. This suggests that a comprehensive understanding of the mechanisms by which ID/IDA impairs brain function needs to consider effects both at the level of neurotransmitter function and at the level of oxygen transport.

All of these results were obtained in the context of the first study to document the efficacy of providing an iron-biofortified grain as a regular dietary component on the iron status of both male and female adolescents. This pattern of results conceptually replicates previous work [35], in which we demonstrated that consumption of a bean, biofortified for increased iron, was capable of producing improvements in blood iron biomarkers, behavioral measures of cognition, and EEG measures of brain function in college-aged women in Rwanda. This suggests that the efficacy of using a dietary intervention to address ID/IDA extends beyond benefits to the body to benefits to brain function and cognition, and is consistent with the outcomes of other studies that have examined the effects of iron depletion and repletion in children, adolescents, and women of reproductive age [6, 10, 11, 45, 47]. It also highlights the importance of biofortification as an intervention, in that it is low in dosage, targeted to the most needy, sustainable, and efficacious at numerous levels of measurement.

The strengths of the present effort include the fact that it was conducted as a randomized, double-blind controlled study. Furthermore, as it concerned the effects of the dietary intervention on functional outcomes that are determined by brain function, it incorporated measures of blood, brain function, and behavior. Additionally, it used cognitive measures that have a long history and rich literature in laboratory work on basic cognition and that were selected based on their dependence on brain regions that are differentially dependent on iron. Finally, it applied statistical methods that allow for assessment of the mediation hypothesis and that are flexible enough to explicitly include hypothesized modifying influences.

The weaknesses of the effort include the fact that the conditions under which the EEG was recorded were far from ideal. Recording was not done in electromagnetically-shielded rooms, and the power source for recording was somewhat unreliable and electrically noisy. As a consequence, even with extensive pre-processing, there was still loss of data. In addition, the EEG data as collected cannot speak to the range of possible mechanisms supporting the changes in the behavioral measures. Although the mediation models provide suggestive evidence of a causal link, they can only be weakly interpreted as evidence for causality. Data were not available on menstrual cycle timing in female participants, and this may have influenced blood biomarker levels; however, randomization should have addressed this concern. Selecting participants based on their sFt levels limits the generality of the results. Finally, we should note that biofortification is but one way of achieving the types of improvements documented here. Alternative strategies to improve iron absorption and status would include dietary modifications and increased dietary diversity, alone or in concert with biofortification.

## Conclusion

In sum, we have provided evidence that consumption of a iron-biofortified pearl millet for six months leads to improvements in measures of iron status, brain function, and cognition. These results add to the increasing evidence that biofortification is an effective method for addressing micronutrient deficiencies and to the evidence that iron repletion by way of a nutritional intervention other than supplementation has measurable and important effects on brain and cognitive function. Furthermore, the inclusion of measures of brain function provide evidence important in motivating future work on the brain mechanisms responsible for supporting changes in cognition and their inherent plasticity.

## Supplementary Information

**Additional file 1** Additional file 1 contains the baseline values of the iron biomarkers, separated by sex; procedural details for each of the cognitive tasks; and the details of the pre-processing of the EEG data.

## Data Availability

Data, task stimuli, and task code are available from MJW on reasonable request.

## Abbreviations

AGP: *α*-1 acid glycoprotein
ANCOVA: Analysis of covariance
ANOVA: Analysis of variance
ANT: Attentional network task
BdFe: Body iron
BPM: Biofortified pearl millet
CFE: Composite face effect
CPM: Comparison pearl millet
CRP: C-reactive protein
CRT: Cued recognition task
DA: Dopamine
DAT: Dopamine transporter
EEG: Electroencephalography
GNG: Go/no-go
Hb: Hemoglobin
ID: Iron deficiency
IDA: Iron deficiency anemia
RT: Reaction time
sFt: Serum ferritin
IDNA: Iron deficient non-anemic
SRT: Simple reaction time
sTfR: Soluble transferrin receptor

## Footnotes

1 The two other general aspects of brain function that can be affected by variations in iron levels are myelination and neurogenesis/synaptogenesis [30–33]. However, to our knowledge there is no evidence in animal models of changes in white-matter integrity or changes in brain morphology.

2 Note that sFt and sTfR are combined in calculating BdFe, and each assess different aspects of bodyn iron status (liver store vs. tissue iron demand, respectively). Consequently, BdFe as a composite measure is a more general indicator of body iron status.
