# Correction: Modeling relationships between iron status, behavior, and brain electrophysiology: evidence from a randomized study involving a biofortified grain in Indian adolescents

**DOI:** 10.1186/s12889-022-13897-0

**Published:** 2022-09-02

**Authors:** Michael J. Wenger, Laura E. Murray Kolb, Samuel P. Scott, Erick Boy, Jere D. Haas

**Affiliations:** 1 Department of Psychology, Cellular and Behavioral Neurobiology, The University of Oklahoma, Norman, OK USA; 2 Division of Nutritional Sciences, Cornell University, Ithaca, NY USA; 3 Department of Nutrition Science, Purdue University, West Lafayette, IN USA; 4 Poverty Health and Nutrition Division, International Food Policy Research Institute, Washington, DC USA; 5 HarvestPlus, International Food Policy Research Institute, Washington, DC USA

**Correction: BMC Public Health 22, 1299 (2022)**

**https://doi.org/10.1186/s12889-022-13612-z**

In the original publication [1] there was an incorrect funding acknowledgement. In this correction article the correct and incorrect funding acknowledgement are published. The original article has been updated.

**Incorrect funding**

- Funding for this project was provided by HarvestPlus, International Food Policy Research Institute.

**Correct funding**

- Financial support for this study was provided by HarvestPlus (www.HarvestPlus.org), a global program working to develop and promote biofortified food crops that are rich in vitamins and minerals needed for good health. HarvestPlus’ principal donors are the UK government and the Bill & Melinda Gates Foundation. The views expressed do not necessarily reflect those of HarvestPlus.
