# Supplementary material for: Modeling relationships between iron status, behavior, and brain electrophysiology: evidence from a randomized study involving a biofortified grain in Indian adolescents
Source: BMC Public Health. 2022 Jul 6;22:1299. doi: 10.1186/s12889-022-13612-z (PMC9260997; doi:10.1186/s12889-022-13612-z)
Supplement: Supplementary file 1 — Additional file 1 Additional file 1 contains the baseline values of the iron biomarkers, separated by sex; procedural details for each of the cognitive tasks; and the details of the pre-processing of the EEG data. [file 12889_2022_13612_MOESM1_ESM.pdf]

Supplementary information

Descriptive statistics

Supplementary Table 1 presents the baseline characteristics of the sample along with tests of the comparisons for treatment condition and sex.

Procedural details of the cognitive tasks

Simple reaction time task (SRT): The simple reaction time task provides an estimate of the simplest possible behavioral response to a visual stimulus, in that the task required the participant to press a button in response to the onset of the task stimulus. The task requires no discrimination or decision-making, has limited attentional and no mnemonic demands. The task stimulus (Supplementary Figure 1) was presented a total of 20 times. The interstimulus interval (ISI) varied on each trial for each participant and was determined using a uniform distribution running from between 750 and 1500 ms. Participants were instructed to press the / key on the computer keyboard as soon as they perceived the stimulus onset. Five practice trials preceded the test trials; feedback was provided only on the practice trials.

Go/No-Go (GNG) task: The go/no-go task assesses simple sustained attention and response control. Stimuli were a horizontal and vertical white bar, centered on a black background, presented on screen. Stimuli were randomly, across both subjects and assessments, assigned to be either the go or the no-go stimulus. Subjects were instructed to press the / key with the index finger of their dominant hand on trials on which the go stimulus was presented, and to withhold a response on trials on which the no-go stimulus was presented.

Prior to beginning the task, participants completed 10 practice trials, consisting of a random ordering of 2 go and 8 no-go trials, each preceded by a centrally presented fixation cross. The exposure duration for the fixation cross was randomly determined on each presentation by a value drawn from a uniform distribution over the interval from 400 to 700 ms. The stimulus was then presented for 300 ms. If participants did not respond to the stimulus within 1700 ms, the sequence moved on to the next fixation and stimulus. The experimenter provided verbal feedback on trials on which the subject made either an error of commission or omission; note that this feedback was provided only on the practice trials. Following the practice trials, the test trials were presented, consisting of a random ordering of 30 go trials and 120 no-go trials, using the timings that were used on the practice trials.

Attentional Network Task (ANT): The attentional network task is intended to probe three functions of attention: low-level attentional capture, mid-level spatial selective attention, and high-level control in the context of information that is nominally irrelevant to the performance of the task. The ANT was implemented according to the description in Fan et al. [1] and as such was run as a 5 (cue type: none, center, double, top, bottom) × 2 (target direction: left, right) × 3 (flanker type: neutral, congruent, incongruent) factorial design with all factors manipulated within-participants, and with six repetitions at each of the combinations of the factor levels. Order of presentation was determined randomly for each participant in each testing session.

Each trial of the task was initiated by the experimenter and began with a fixation cross presented in the center of the screen. The fixation duration was determined randomly on each trial using a value drawn from a uniform distribution over the interval running from 300 to 600 ms. The fixation cross was then replaced by a cue (an asterisk) in the appropriate location, with the cue being present for 200 ms. This was then replaced by a second display of the fixation cross for 200 ms, followed by the test stimulusa left- or right-pointing arrow that was presented among a set of flanking elements, either above or below fixation (Supplemental Figure 1)which was present for up to 1500 ms, or until the participant responded. Participants indicated their judgment of the direction of the target arrow using the index fingers of their left and right hands, pressing the "z" or "/" keys. The test trials were preceded by five

Supplementary Table 1: Baseline characteristics of the sample and comparisons across conditions. Means (standard errors) and Ns (percentage) are reported for males and females in both treatment conditions, along with F-statistics for the two-way ANOVA and χ^2 statistics for the tests of association. Note: $+ = 0.05 <= p < 0.10, * = p < 0.05, ** = p < 0.01$, CPM = comparison pearl millet, BPM = biofortified pearl millet.

		CPM		BPM			
	Overall n = 74	Male n = 17	Female n = 16	Male n = 18	Female n = 23	Condition	Sex
						F, Main Effects	
Age (y)	13.5 (0.2)	13.4 (0.2)	13.8 (0.4)	13.5 (0.3)	13.4 (0.3)	0.22	0.41
Hb, g/dL	12.13 (0.13)	12.46 (0.23)	11.89 (0.31)	12.47 (0.19)	11.80 (0.29)	0.11	5.78*
sFt, ng/mL	15.41 (1.56)	11.83 (1.51)	15.01 (3.11)	17.54 (3.01)	16.66 (3.73)	1.37	0.09
sTfR, ug/mL	7.15 (0.38)	7.20 (0.79)	7.51 (1.21)	7.22 (0.75)	6.80 (0.54)	0.22	0.01
BdFe (mg/kg)	0.61 (0,.37)	0.06 (0.61)	0.58 (0.75)	1.27 (0.70)	0.53 (0.82)	0.52	0.05
Prevalences						χ^2, Association	
Anemia	26 (35)	2 (12)	9 (56)	4 (22)	11 (48)	0.08	9.43**
sFt < 15.0	46 (62)	13 (76)	10 (63)	9 (50)	14 (61)	1.44	0.01
sTfR > 8.3	11 (15)	2 (12)	1 (6)	6 (33)	2 (9)	1.57	3.35+
BdFe < 0.0	26 (35)	8 (47)	4 (25)	5 (28)	9 (39)	0.04	0.12
IDA	16 (22)	2 (12)	6 (38)	1 (6)	7 (30)	0.24	6.67**
IDNA	30 (41)	11 (65)	4 (25)	8 (44)	7 (30)	0.60	5.21*
Inflammation	4 (5)	0 (0)	1 (6)	1 (6)	2 (9)	0.66	0.84

practice trials (selected randomly for each participant in each session). The practice trials differed from the test trials only in that they were followed by feedback on the participants response.

Composite Face Effect (CFE) task: The composite face effect task assesses the extent to which semantic memory can influence visual selective attention, the highest level of attention assessed in the ANT. The effect involves the presentation of facial stimuli (Figure 1) in which the top and bottom portions are drawn from either the same or different faces, and in which those two portions are either aligned or misaligned. The critical comparison in this task involves stimuli in which the top and bottom portions are drawn from familiar (well known, e.g., celebrities) vs. unfamiliar faces. The canonical effect, indexing the influence of semantic memory on selective attention, is that identification of one half of a target face is impaired (slowed, possibly with lower accuracy) when the two portions of the face are aligned relative to when they are mis-aligned, with this performance decrement obtaining only when the top and bottom portions are drawn from two familiar faces. The CFE was implemented as a 2 (familiarity: unfamiliar, familiar) $\times$ 2 (identity of the top portion: person 1, person 2) $\times$ 2 (identity of the bottom portion: person 1, person 2) $\times$ 2 (target portion to be identified: top, bottom) $\times$ 2 (alignment: unaligned, aligned) complete factorial design, with all factors manipulated within participants. The two familiar faces were female Bollywood actresses, judged to be easily identified by participants by the local research staff. The two unfamiliar faces were female members of the Indian National Academy of Sciences, of approximately the same age as the two familiar individuals. Test trials were blocked by familiarity, with familiar faces tested before unfamiliar faces.

Each block of trials began with the training of identity responses. The two identities (either the two familiar or two unfamiliar faces) were randomly and with equal likelihood assigned to an "A" (person 1) or "B" (person 2) response for each participant in each session. Participants were trained in each block to identify each source face to a criterion level of experience. Each block of trials began with the presentation of the two source faces, along with the paired response key (either the "z" or the "/" key, determined randomly and with equal likelihood for each participant at each time point). Ten practice trials were then presented, in which each of the source identities was presented (order was determined randomly), an identity response was required, and feedback was presented.

The test trials were each initiated by the experimenter. Each trial began with a centrally-presented fixation cross, with a duration determined randomly on each trial by a value drawn from a uniform distribution over the interval from 300 to 700 ms. This was then replaced by the test stimulus: a face in one of the possible conditions with a set of three asterisks immediately to the right of the portion (top or bottom) that was to be identified. Ten practice trials, drawn randomly from the set of possible test trials in each block at each time point for each participant, preceded each block of test trials.

Cued recognition task (CRT): The cued recognition task is a variation on the classic recognition memory paradigm, in which an individual is presented with a set of items to be remembered, and is then tested on those items and an equal number of previously unseen items, and asked (for each test item) for a judgment as to whether the item was previously seen (an "old" item) or not (a "new" item). Critically, this task was included because of evidence suggesting that decrements in the amount of work that can be accomplished during recognition is correlated with the level of compromise to neural circuitsparticularly those involving the hippocampusthat support recognition memory.

The CRT was implemented as a 2 (test item type: old, new) $\times$ 3 (number of cues at test: 2, 3, 4) factorial design, with all factors manipulated within participants. There were four trials at each possible combination of factors. Stimuli (Figure 1) were a set of gray scale images, obtained from two sources, the first being a standardized and normed set of images created for use in recognition memory tasks and the second being a set of picture vocabulary images in use in a school in a nearby city. All images were judged to be easily identifiable, name-able, and culturally appropriate by local research staff.

The task was performed in two phases. The first was a study phase, in which participants were told that they would be seeing a set of pictures, and that the task was to study them in advance of a memory test that would follow. The complete set of study trials was initiated by the experimenter. The set of images was then presented, randomized for each participant in each session, at a rate of 3

s/image. The experimenter then gave the instructions for the test trials, which were initiated by the experimenter. Each trial consisted of the presentation of a test image (either an old or new item) with the appropriate number of cues (2, 3, or 4). The recognition cues were the number of equally-sized quadrants of the image that were visible. This was done by dividing the images into four 300 200 pixel quadrants. A random set of the appropriate number of quadrants (determined for each subject on each trial in each session) was covered by a black square. Participants were instructed to give a positive recognition judgment ("old") using the index finger of their right hand on the "/" key of the computer keyboard, and a negative recognition judgment ("new") using the index finger of their left hand on the z key. Participants were instructed to respond as quickly and as accurately as possible, and feedback was not provided.

EEG data pre-processing

The raw EEG data for each individual subject were prepared for statistical analyses in the following way. All pre-processing was done using EEGLAB, an open-source EEG analysis toolbox [2]. First, the data were visually inspected and noisy or otherwise obviously problematic channels were identified and deleted. The data were then subjected to automated procedures for the detection of artifacts, including motion artifacts and blinks. The data were then filtered using a 1 to 90 Hz bandpass filter along with a 50 Hz notch filter. The data were segmented with respect to the onset of the task stimulus, retaining a 500 ms pre-stimulus baseline period and a 1,200 ms post-stimulus processing period. Next, an independent components analysis was performed, and components associated with artifacts and non-brain activity were identified and deleted automatically. Following this, the global field power [3] for the data was estimated, as the basis for identifying critical features in the event-related potentials. Three canonical ERP features were selected for each task: (a) the P1, a positive-going feature, occurring approximately 100 ms following the onset of the task stimulus, reflecting initial perceptual processing; (b) the N1, a negative-going feature, occurring between 150 and 250ms, reflecting object-level identification and processing; and (c) the P3, a positive-going feature occurring between 300 and 500ms following the onset of the task stimulus, reflecting semantic processing [4]. In order to identify these features, the peaks in the global field power within these times windows was first identified. The timing of these peaks in the global field power was then used to select the electrode having the greatest amplitude within an 80 ms window centered on the time of the peak in the global field power. The peak amplitude and the time from the onset of the stimulus to this peak were then retained as variables for analysis. In addition, a Fourier analysis was performed on the segments, and normalized spectral power for the α-(8-15 Hz) and γ-bands (30-90 Hz) was obtained for each electrode.

References

[1] Fan, J., McCandliss, B.D., Sommer, T., Raz, A., Posner, M.I.: Testing the efficiency and independence of attentional networks. Journal of Cognitive Neuroscience **14**(3), 340–347 (2002)

[2] Delorme, A., Mackeig, S.: EEGLab: an open source toolbox for analysis of single-trial EEG dynamics including independent component analysis. Journal of Neuroscience Methods **134**, 9–21 (2004)

[3] Skrandies, W.: Data reduction of multichannel fields: Global field power and principal components analysis. Brain Topography **2**, 73–80 (1989)

[4] Woodman, G.F.: A brief introduction to the use of event-related potentials in studies of perception and attention. Attention, Perception, & Psychophysics **72**(8), 2031–2046 (2010)

Task	*Example stimulus*	*Dimensions (deg. visual angle)*
Simple reaction time (SRT)	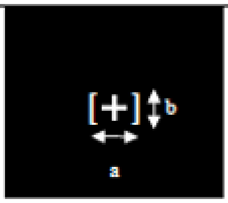	a = 1.06<br>b = 1.74
Go/No-Go (GNG)	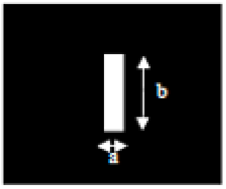	a = 0.10<br>b = 1.20
Attentional network (ANT)	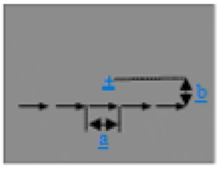	a = 1.10<br>b = 0.80
Composite Face Effect (CFE)	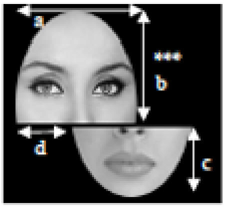	a = 3.11<br>b = 2.62<br>c = 1.72<br>d = 1.55
Cued recognition (CRT)	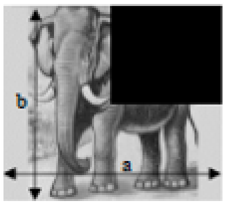	a = 12.09<br>b = 8.29

Supplementary Figure 1: Example stimuli. Images for the composite face effect task were obtained by way of searches on Google Images.
